# Supplementary material for: Social relations and mental ill-health among newly arrived refugees in Sweden: A cross-sectional study
Source: PLOS Glob Public Health. 2022 Jul 7;2(7):e0000362. doi: 10.1371/journal.pgph.0000362 (PMC10021175; doi:10.1371/journal.pgph.0000362)
Supplement: Attachment — Submitted filename: Comments for editor 220321.docx [file pgph.0000362.s001.docx]

1. Your co-authors, Mathias Grahn ([mathias.grahn@malmo.se](mailto:mathias.grahn@malmo.se)), Slobodan Zdravkovic ([slobodan.zdravkovic@mau.se](mailto:slobodan.zdravkovic@mau.se)), Carin Cuadra ([carin.cuadra@mau.se](mailto:carin.cuadra@mau.se)), have not confirmed authorship of the manuscript. We have resent them the authorship confirmation email; however please check that the above email address for them is correct and follow up personally to ensure they confirm. Please note that we cannot pass your manuscript to Production until we have received confirmations from all co-authors.

Answer: This is now done

2. Please include additional information regarding the survey or questionnaire used in the study and ensure that you have provided sufficient details that others could replicate the analyses. For instance, if you developed a questionnaire as part of this study and it is not under a copyright more restrictive than CC-BY, please include a copy, in both the original language and English, as Supporting Information.

Answer: The questionnaire is developed by the research group and the results are published in a report in Swedish file:///C:/Users/ab2389/Downloads/MILSA%201%20KartlC3A4ggning%20av%20nyanlC3A4ndas%20hC3A4lsa.pdf

. Since the questionnaire is developed by different researchers, we are not allowed to share this questionnaire. So far, we have never shared the questionnaire with any journals.

3. Please update your Competing Interests statement. If you have no competing interests to declare, please state: “The authors have declared that no competing interests exist.”

Answer: this is now added.

4. Please amend your detailed Financial Disclosure statement. This is published with the article, therefore should be completed in full sentences and contain the exact wording you wish to be published.

i). State the initials, alongside each funding source, of each author to receive each grant.

ii). State what role the funders took in the study. If the funders had no role in your study, please state: “The funders had no role in study design, data collection and analysis, decision to publish, or preparation of the manuscript.”

Answer: This is added

5. In the online submission form, you indicated that “Data can be requested from the authors.”. All PLOS journals now require all data underlying the findings described in their manuscript to be freely available to other researchers, either 1. In a public repository, 2. Within the manuscript itself, or 3. Uploaded as supplementary information.

This policy applies to all data except where public deposition would breach compliance with the protocol approved by your research ethics board. If your data cannot be made publicly available for ethical or legal reasons (e.g., public availability would compromise patient privacy), please explain your reasons by return email and your exemption request will be escalated to the editor for approval. Your exemption request will be handled independently and will not hold up the peer review process, but will need to be resolved should your manuscript be accepted for publication. One of the Editorial team will then be in touch if there are any issues.

Answer: we are not allowed to share data and this is edited within the manuscript.
